# Hippo Signaling in the Lung: A Tale of Two Effectors—Yap Drives Airway Fate and Taz Drives Alveolar Differentiation

**DOI:** 10.3390/cells15020143

**Published:** 2026-01-13

**Authors:** Rachel Warren, Stijn P. J. De Langhe

**Affiliations:** 1 Department of Medicine, Division of Pulmonary and Critical Medicine, Mayo Clinic, Rochester, MN 55905, USA; 2 Department of Biochemistry and Molecular Biology, Mayo Clinic, Rochester, MN 55905, USA

**Keywords:** Hippo, lung stem cell, Yap, Taz, cell competition, fibrosis, Myc

## Abstract

**Highlights:**

**What are the main findings?**

- YAP and TAZ exhibit functional non-redundancy in the lung: YAP drives airway progenitor expansion, while TAZ is the obligate driver of alveolar differentiation via an NKX2-1 feed-forward loop.
- The “See-Saw” and “Niche Collapse” models explain that “honeycombing” can be triggered by a loss of mesenchymal fitness, which releases a mechanical brake on the epithelium.

**What is the implication of the main finding?**

- Classification of Small Cell Lung Cancer (SCLC) subtypes through a developmental lens identifies the YAP1+ (SCLC-Y) state as a “supercompetitor” that mirrors maladaptive regenerative states.
- Therapeutic strategies for fibrosis must move beyond anti-inflammatory approaches to focus on restoring niche fitness (e.g., Snail stabilization) and inhibiting the YAP-driven “supercompetitor” state.

**Abstract:**

The mammalian lung operates under a biological paradox, requiring architectural fragility for gas exchange while maintaining robust regenerative plasticity to withstand injury. The Hippo signaling pathway has emerged as a central “rheostat” in orchestrating these opposing needs, yet the distinct roles of its downstream effectors remain underappreciated. This review synthesizes recent genetic and mechanobiological advances to propose a “Tale of Two Effectors” model, arguing for the functional non-redundancy of YAP and TAZ. We posit that YAP functions to drive airway progenitor expansion, mechanical force generation, and maladaptive remodeling. Conversely, TAZ—regulated uniquely via transcriptional mechanisms and mechanotransduction—acts as an obligate driver of alveolar differentiation and adaptive repair through an NKX2-1 feed-forward loop. Furthermore, we introduce the “See-Saw” model of tissue fitness, where mesenchymal niche collapse releases the mechanical brake on the epithelium, triggering the bronchiolization characteristic of pulmonary fibrosis. Finally, we extend this framework to malignancy, illustrating how Small Cell Lung Cancer (SCLC) subtypes mirror these developmental and regenerative states. This integrated framework offers new therapeutic distinct targets for modulating tissue fitness and resolving fibrosis.

## 1. Introduction: The Paradox of Pulmonary Plasticity

The mammalian lung exists in a state of perpetual physiological contradiction, a biological paradox that challenges the fundamental definitions of structural stability and cellular plasticity. Morphologically, the lung acts as a delicate, high-surface-area interface required for gas exchange, possessing a blood-gas barrier often thinner than a single micron [1]. This architectural fragility is a prerequisite for its primary function: the efficient diffusion of oxygen and carbon dioxide. Yet, this structural delicacy is juxtaposed against a robust, facultative regenerative capacity that allows the organ to rebuild its complex architecture following catastrophic injury, such as viral pneumonia, chemical insult, or bacterial sepsis. The lung must be rigid enough to withstand the mechanical strain of millions of breath cycles per lifetime, yet plastic enough to mobilize stem cell pools and reconstruct alveoli when the barrier is breached [2].

The orchestration of these opposing necessities—the maintenance of structural stability during homeostasis and the activation of plastic regenerative programs during repair—relies on a sophisticated, integrated network of molecular signaling pathways. Among these, the Hippo signaling pathway and its downstream transcriptional effectors, yes-associated protein (YAP) and transcriptional co-activator with PDZ-binding motif (TAZ, encoded by *WWTR1*), have emerged as the central “rheostat” of pulmonary biology [3]. Unlike canonical signaling pathways such as Wnt, BMP, or Notch, which typically rely on dedicated extracellular ligands binding to transmembrane receptors to initiate a linear signal transduction cascade, the Hippo pathway functions as a decentralized sensor of the cellular microenvironment. It integrates a diverse and often noisy array of inputs, including cell–cell contact (contact inhibition), mechanical tension, extracellular matrix (ECM) stiffness, energy status (glucose availability), and G-protein coupled receptor (GPCR) signaling, to dictate cell fate decisions with exquisite precision [2].

The evolutionary conservation of this pathway, from *Drosophila melanogaster* where it was first identified as a regulator of organ size, to mammals where it governs stem cell potency and tissue regeneration, underscores its fundamental importance. However, in the complex multicellular environment of the mammalian lung, the pathway has evolved beyond simple size control. It has become a master integrator of “tissue fitness,” assessing the competitive viability of cells within their niche [4]. This comprehensive report synthesizes a vast body of recent high-fidelity genetic studies, transcriptomic analyses, and mechanobiological investigations to present an integrative framework of Hippo signaling in the lung.

We move beyond the canonical view of the pathway as a simple regulator of organ size to explore its role as a complex integrator of mechanical tension, metabolic status, and cell competition. A central thesis of this review is the functional non-redundancy of the effectors: we propose a “Tale of Two Effectors” model wherein YAP functions as the driver of airway progenitor expansion and, pathologically, maladaptive remodeling (the “supercompetitor”), while TAZ acts as the obligate driver of alveolar differentiation and adaptive repair (the “loser” or differentiator) [5,6].

## 2. Genomic and Structural Architecture: The Foundation of the Hippo Network

To understand the specific roles of Hippo signaling in the lung, one must first dissect the molecular machinery that governs it. The pathway is a dynamic network with layers of regulation ranging from protein stability to chromatin topology. The precision of pulmonary development and repair depends on the ability of this network to filter noise and transduce specific environmental cues into distinct transcriptional outputs.

### 2.1. The Core Kinase Cassette and Effector Regulation

In mammals, the canonical core of the pathway consists of a kinase cascade that functions essentially as a “brake” on cellular growth and proliferation. The upstream kinases MST1 and MST2 (STK4/3) phosphorylate and activate the downstream kinases LATS1 and LATS2 (Large tumor suppressor kinase 1/2). This activation is facilitated by the scaffolding proteins SAV1 (Salvador) and MOB1 (Mps One Binder), which organize the kinase complexes at specific subcellular locations, primarily the apical membrane and cell–cell junctions [3]. The phosphorylation cascade culminates in the regulation of the transcriptional co-activators YAP and TAZ. Once activated, LATS1/2 phosphorylate these effectors at specific serine residues—notably Serine 127 on YAP and Serine 89 on TAZ [7,8]. This phosphorylation event generates high-affinity binding motifs for 14-3-3 proteins. The binding of 14-3-3 leads to the cytoplasmic sequestration of YAP/TAZ, effectively “caging” them away from the nucleus and preventing access to the genome.

**Figure 1:**
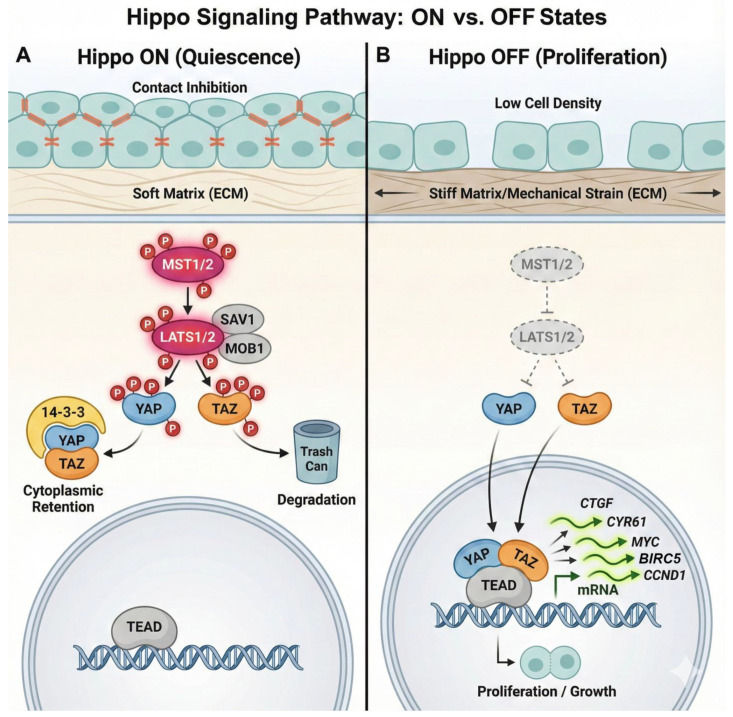
**General overview of the canonical Hippo signaling pathway inputs, regulation, and outputs.** (**A**) Hippo ON (Quiescence). Under conditions of high cell density (contact inhibition), soft extracellular matrix (ECM), or nutrient deprivation, the Hippo pathway is active. The core kinase cassette, consisting of MST1/2 and LATS1/2 (facilitated by scaffolds SAV1 and MOB1), acts as a “brake” on cellular growth. Activated LATS1/2 phosphorylates the effectors YAP and TAZ at specific serine residues. This phosphorylation promotes binding to 14-3-3 proteins, leading to cytoplasmic sequestration (“caging”), or primes them for ubiquitination and proteasomal degradation. Consequently, YAP and TAZ are excluded from the nucleus, and the cell remains quiescent. (**B**) Hippo OFF (Proliferation). In response to mechanical strain, stiff ECM, or loss of cell–cell contact, the kinase cassette is inactivated (“Hippo off”). YAP and TAZ remain unphosphorylated and rapidly translocate into the nucleus. Once nuclear, they function as transcriptional co-activators, partnering with TEAD family transcription factors. This complex binds to enhancers to drive the expression of target genes essential for cell proliferation (*MYC*, *CCND1*) and matricellular protein production (*CTGF*, *CYR61*). Created with Nano Banana Pro.

This sequestration is reversible, allowing the cell to maintain a pool of ready-to-deploy effectors. However, further phosphorylation by kinases such as CK1 kinases can prime YAP/TAZ for recognition by the β transducing repeat-containing protein β-TrCP, an adapter for the SCF E3 ubiquitin ligase complex, leading to their polyubiquitination and irreversible proteasomal degradation [9]. Thus, when the Hippo pathway is “on”—typically in conditions of high cell density (contact inhibition), soft extracellular matrix (ECM), or nutrient deprivation—YAP and TAZ are inhibited, and the cell remains quiescent [2]. Conversely, when the pathway is “off”—due to mechanical strain, specific growth factor inputs (e.g., LPA, S1P signaling via GPCRs), or loss of cell contact—YAP and TAZ remain unphosphorylated (Figure 1). In this state, they rapidly translocate to the nucleus. Since YAP and TAZ lack intrinsic DNA-binding domains, they act as transcriptional co-activators, partnering with DNA-binding transcription factors to exert their effects. In the lung, their primary and most potent partners are the TEAD family of transcription factors (TEAD1–4). The YAP/TAZ-TEAD complex binds to specific enhancer regions to drive distinct gene expression programs essential for proliferation (*CCND1*, *MYC*), survival (*BIRC5*), and the production of matricellular proteins (*CTGF*, *CYR61*). This output is highly context-dependent, relying on the availability of other co-factors and the chromatin landscape of the specific cell lineage [10].

### 2.2. Genomic Architecture of WWTR1 (TAZ)

While the regulation of YAP is often discussed in terms of protein stability (phosphorylation and degradation), emerging evidence underscores that the transcriptional regulation of the WWTR1 gene (which encodes TAZ) represents a critical, independent layer of control. Unlike a constitutive housekeeping gene, WWTR1 is dynamically regulated by a convergence of oncogenic signaling, mechanotransduction, and metabolic sensors. Despite the presence of a phosphodegron, endogenous YAP is a relatively stable protein, mainly regulated by nuclear-cytoplasmic shuttling. In contrast, TAZ is a very unstable protein with a half-life of <2 h, indicating that protein degradation is the main route for TAZ inhibition [9]. Understanding this genomic architecture is prerequisite to understanding why TAZ operates distinctively from YAP in the pulmonary context [11].

#### 2.2.1. Promoter Architecture: The CpG-Rich, TATA-Less Landscape

The transcriptional output of WWTR1, located on human chromosome 3q25.1, is determined fundamentally by its unique promoter structure. Detailed sequence analysis indicates that the WWTR1 core promoter lacks a canonical TATA box, a cis-regulatory element typically associated with precise transcription start site (TSS) selection in tissue-specific genes. The absence of a TATA box necessitates alternative mechanisms for the assembly of the pre-initiation complex (PIC). Instead of TATA elements, the WWTR1 regulatory region is characterized by a high density of CpG dinucleotides. These CpG islands are not randomly distributed but are concentrated in specific regulatory zones (TAZ-1, TAZ-2, and TAZ-3) flanking the transcription start site [12]. The functional implication of this architecture is a high susceptibility to regulation via DNA methylation. In normal physiological states, these islands generally remain unmethylated to permit the binding of GC-box binding factors, such as Sp1, which often anchor the transcriptional machinery to TATA-less promoters. However, this high GC content renders the gene vulnerable to epigenetic silencing through hypermethylation. This likely acts as a fail-safe mechanism for tumor suppression in differentiated tissues where constitutive TAZ activity would be deleterious.

#### 2.2.2. Super-Enhancers and Chromatin Topology

Beyond the proximal promoter, the transcriptional magnitude of WWTR1 is heavily influenced by distal cis-regulatory elements known as super-enhancers (SEs). These large clusters of transcriptional enhancers are densely occupied by master transcription factors, Mediator complexes, and chromatin readers, driving high-level expression of genes that define cell identity. Recent genome-wide H3K27ac (Histone H3 lysine 27 acetylation) profiling has revealed that WWTR1 is controlled by prominent super-enhancers in specific oncogenic and developmental contexts [10].

A striking example of this regulation is found in neuroblastoma models that oscillate between a Mesenchymal (MES) state and an Adrenergic (ADRN) state [13]. In this context, the *WWTR1* locus is a hallmark target of the MES-specific super-enhancer landscape. In MES cells, the *WWTR1* locus is marked by broad H3K27ac domains and is bound by MES-specific master regulators such as PRRX1, SNAI2, and crucially, AP-1 transcription factors (FOS/JUN). The involvement of AP-1 is particularly relevant to lung injury repair, as AP-1 factors are central regulators of the “transitional” epithelial state, often termed the DATP (Damage-Associated Transient Progenitor) or PATS (Pre-Alveolar Type 1 Transitional State) [14]. This suggests a conserved regulatory logic where TAZ expression is boosted by super-enhancers to drive mesenchymal or transitional states characterized by high plasticity and migration.

### 2.3. Mechanotransduction at the Transcriptional Level: The SRF/MRTF Axis

TAZ is famously known as a mechanotransducer at the protein level (via nuclear shuttling), but its transcription is also mechanically regulated, providing a sustained response to physical cues, a feature particularly relevant in the stiffened matrix of fibrotic lungs. The WWTR1 promoter contains functional CArG box motifs (CC(A/T)6GG), which are the canonical binding sites for the Serum Response Factor (SRF). The ability of SRF to drive WWTR1 transcription is dependent on Myocardin-related transcription factors (MRTF-A and MRTF-B, also known as MKL1/2) [3,15].

**Figure 2:**
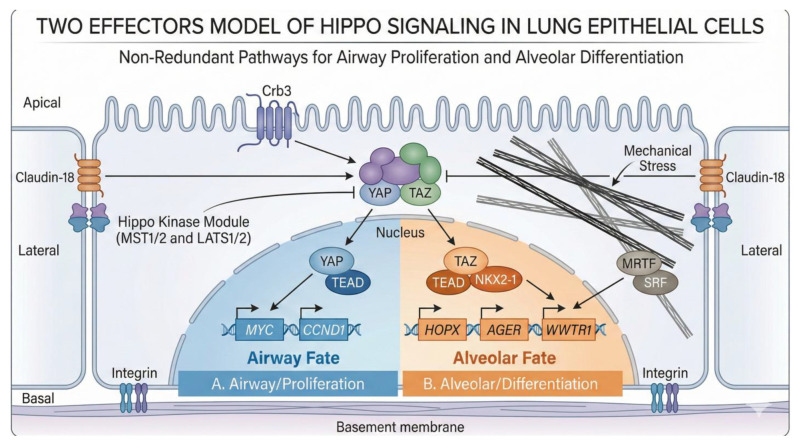
**The “Two Effectors” model of Hippo signaling non-redundancy in the lung.** This schematic illustrates the divergent downstream fates of the Hippo pathway effectors YAP and TAZ. While the upstream kinase module (MST1/2, LATS1/2) and polarity proteins (Crb3, Claudin-18) universally function as a “brake” on nuclear entry, the nuclear output is distinct. (**Left**) YAP (blue) pairs with TEAD to drive an “Airway” program characterized by proliferation markers (*MYC*, *CCND1*), typical of high-fitness progenitors. (**Right**) TAZ (orange) acts as the obligate driver of the “Alveolar” program. TAZ transcription is independently regulated by cytoskeletal tension via the MRTF/SRF axis and forms a feed-forward loop with NKX2-1 to drive Alveolar Type 1 (AT1) differentiation genes (*HOPX*, *AGER*) and its own expression (*WWTR1*). Take-home message: YAP and TAZ are not redundant; YAP drives airway fate and proliferation, while TAZ is required for alveolar differentiation. Created with Nano Banana Pro.

In soft environments, MRTFs are sequestered in the cytoplasm by monomeric G-actin. Upon mechanical stiffening or cytoskeletal tension (which induces F-actin polymerization), the pool of G-actin is depleted, releasing MRTFs from cytoplasmic sequestration. MRTFs then translocate to the nucleus, where they associate with SRF at the *WWTR1* promoter to drive robust transcription (Figure 2). This creates a powerful feed-forward loop: stiff matrices promote TAZ transcription via MRTF/SRF. TAZ protein then drives the expression of ECM components and cytoskeleton regulators (like *Acta2* and *Cyr61*), further increasing intracellular tension and matrix stiffness [15]. This mechanotransduction is further amplified by Discoidin Domain Receptor 1 (DDR1), a collagen receptor. DDR1 signaling promotes MRTF nuclear accumulation, enhancing WWTR1 transcription, while TAZ feeds back to upregulate DDR1 [16]. This stiffness-sensing positive feedback loop effectively locks cells into a “mechanospite” phenotype, a key driver of fibrosis where the cell becomes biologically programmed to perpetuate the stiff environment it inhabits [17].

## 3. Architectural Foundations: Hippo Signaling in Lung Morphogenesis

The establishment of the functional lung requires the generation of a branched airway tree (conducting zone) terminating in millions of alveoli (respiratory zone). This architecture is laid down during embryogenesis through a tightly regulated process of branching morphogenesis, which relies on the precise spatial coordination of epithelial proliferation and differentiation. The Hippo pathway acts as a master integrator during these stages, orchestrating the physical sculpting of the organ.

### 3.1. Branching Morphogenesis and Mechanical Force Generation

The pseudoglandular stage of lung development (E9.5–E16.5 in mice) is characterized by repetitive epithelial bifurcation and invasion into the surrounding mesenchyme. YAP is indispensable for this process. Conditional deletion of Yap (using the *Shh-Cre* driver) in the early lung epithelium results in severe lung hypoplasia and a complete arrest of branching morphogenesis [18]. This phenotype is significantly more severe than that observed with *Nkx2.1-Cre* mediated deletion, likely due to the higher recombination efficiency and earlier onset of *Shh-Cre* [19]. Mechanistically, YAP does not function solely as a driver of cell cycle progression in this context. While proliferation is required for growth, the physical act of budding requires force. Transcriptomic and ChIP-seq analyses have revealed that YAP regulates a specific suite of genes involved in cytoskeletal dynamics and cellular contractility, including Arhgef17 (a RhoGEF), Bcam, S1pr2 (sphingosine-1-phosphate receptor), and Nuak2 [20]. These targets modulate the phosphorylation of myosin light chain (pMLC), thereby generating the cortical tension required for epithelial tube deformation and budding [20]. In Yap-deficient lungs, the failure to generate sufficient mechanical force results in an inability to execute the branching program, despite the presence of other growth factors. This establishes YAP as a “mechanochemical” engine of organogenesis [20]. Furthermore, YAP regulates the feedback loops between the epithelium and mesenchyme. In the absence of epithelial YAP, expression of *Shh* is reduced while *Fgf10* is upregulated [19].

### 3.2. The YAP-TAZ Handover: Sequential Requirements

A critical insight derived from comparative genetic models is the temporal distinction between YAP and TAZ requirements. While YAP is essential for the early expansion of the epithelial progenitor pool and branching morphogenesis, TAZ appears to play a subordinate role during these early stages. Wwtr1 (TAZ) knockout mice do not exhibit the severe branching arrest seen in Yap mutants. However, TAZ becomes indispensable during the later stages of development, specifically for alveologenesis and the maturation of the gas-exchange surface [19,21,22]. This suggests a “sequential handover” model: YAP drives the rapid expansion and structural sculpting of the airway tree (pseudoglandular/canalicular stages), whereas TAZ orchestrates the terminal differentiation and physiological maturation of the alveolar compartment (saccular/alveolar stages) (Figure 2). *Wwtr1*-deficient mice develop an emphysema-like phenotype in adulthood, characterized by enlarged airspaces and defective alveolar septation, underscoring TAZ’s specific requirement for alveolar integrity [19,23].

### 3.3. Proximal–Distal Patterning and the Transition Zone

The developing lung epithelium is spatially patterned along a proximal–distal axis, with Sox2+ progenitors generating airways and Sox9+ progenitors generating the distal gas-exchange units during late lung development (during early lung development Sox9 progenitors also give rise to Sox2+ progenitors). The role of YAP in establishing this boundary has been a subject of debate. Early reports suggested a “transition zone” model where nuclear YAP was confined to a specific region between the Sox2+ and Sox9+ compartments, ostensibly to sensitize cells to TGF-β signaling. However, more rigorous immunofluorescence analysis has shown that active, nuclear YAP is distributed throughout the lung epithelium during branching, present in both Sox2+ and Sox9+ populations [19]. The loss of YAP disrupts morphogenesis globally rather than locally at a transition zone. Nevertheless, the subcellular localization of YAP does undergo dynamic shifts. In the distal buds, YAP transitions from a predominantly nuclear state (driving proliferation/expansion) to a pancellular or cytoplasmic state prior to sacculation. Interestingly, while nucleocytoplasmic shuttling is a hallmark of Hippo regulation, it appears that for many developmental processes, the mere presence of nuclear YAP is sufficient. Genetic rescue experiments expressing a constitutively nuclear form of YAP (Yap5SA) in Yap-deficient lungs were able to rescue branching defects. However, the persistence of nuclear YAP in compartments where it should be downregulated leads to pathology. For example, maintained nuclear YAP activity in the proximal airways inhibits the differentiation of basal cells into secretory and ciliated cells, locking them in a basal-like progenitor state [18]. This indicates that while nuclear YAP is sufficient for morphogenesis, its eventual exclusion from the nucleus is strictly required for terminal differentiation.

### 3.4. Polarity, Tight Junctions, and Structural Regulation

The regulation of YAP during development is intimately tied to apicobasal polarity and cell–cell junctions, providing a direct link between tissue architecture and transcriptional control. Signals from distinct cellular domains converge to ensure robust Hippo pathway activation and YAP sequestration.

**Crb3 (Crumbs3):** This polarity protein serves as the guardian of the apical domain. Crb3 promotes the interaction between YAP and LATS1/2 at apical junctions, facilitating YAP phosphorylation and cytoplasmic retention. In the absence of Crb3, YAP accumulates in the nucleus, preventing airway epithelial differentiation and leading to a hyperplastic, progenitor-like phenotype. This provides a direct mechanistic link between the establishment of epithelial polarity and the exit from the cell cycle via Hippo signaling [24] (Figure 2).

**Claudin-18 (CLDN18):** At the lateral domain, the lung-specific tight junction protein CLDN18 restricts YAP activity. CLDN18 interacts with YAP at cell–cell contacts and facilitates its phosphorylation by LATS1/2. Loss of *Cldn18* results in increased nuclear YAP, leading to the expansion of Alveolar Type 2 (AT2) progenitor cells, increased organ size, and a predisposition to lung adenocarcinoma. This demonstrates that structural components of the epithelium act as continuous physiological sensors, coupling tissue architecture to proliferative control via the Hippo pathway [25] (Figure 2).

**Integrin-linked Kinase (ILK):** Completing the polarity circuit at the basal domain, ILK serves as an essential orientation signal for apicobasal polarity. Loss of ILK disrupts the alignment of the apical surface and lumen formation. In the context of Hippo signaling, ILK is required to recruit and stabilize Merlin (NF2) at the membrane. Without this basal anchor, Merlin is degraded, the Hippo pathway is inactivated, and YAP translocates to the nucleus [19] (Figure 2).

### 3.5. The “See-Saw” of Mesenchymal–Epithelial Antagonism

While early studies focused on epithelial YAP, recent work by Klinkhammer et al. (2025) introduces the concept of mechanical antagonism between tissue compartments, fundamentally shifting our understanding of morphogenetic control [4]. During development, high YAP activity in the mesenchyme is required to suppress YAP activity in the epithelium.

The Mechanism: High mesenchymal fitness (driven by Yap/Taz and Snail1/2) restrains epithelial expansion. This suppression allows the epithelium to transition from a proliferative, branching state (high YAP) to a differentiated alveolar state (low YAP).

Loss of Balance: If mesenchymal YAP is genetically deleted (e.g., using *Tbx4-rtTa*; *Tet-Cre*), this suppressive “brake” is removed. Consequently, the epithelium senses this loss of constraint and upregulates its own YAP signaling, failing to differentiate properly and leading to architectural and likely functional defects.

**Figure 3:**
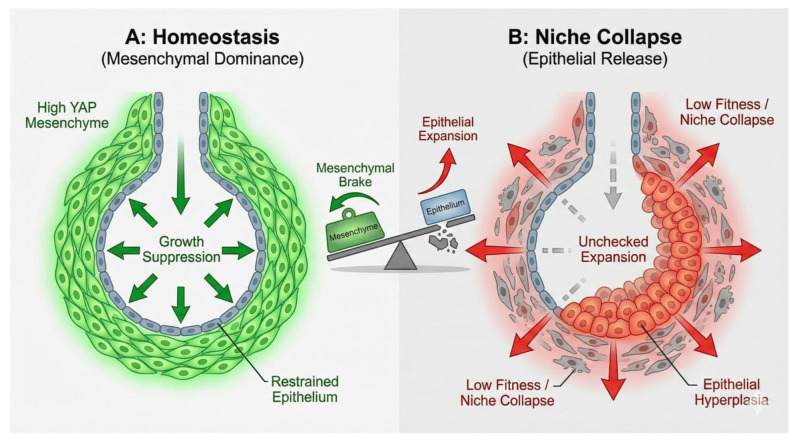
**The “See-Saw” model of tissue fitness and mechanical antagonism.** A conceptual diagram illustrating the mechanical balance between tissue compartments. (**A**) **Homeostasis:** High-fitness mesenchymal cells (green) exert a mechanical constraint (“Growth Suppression”) on the epithelium, maintaining alveolar structure. (**B**) **Niche Collapse:** The loss of mesenchymal fitness (apoptosis) releases this mechanical brake. The epithelium senses this loss of constraint (“Unchecked Expansion”) and activates a compensatory YAP program, leading to hyperplasia and the failure of proper alveolar differentiation. Take-home message: Epithelial bronchiolization can be a secondary consequence of mesenchymal niche collapse (loss of fitness). Created with Nano Banana Pro.

This establishes a “See-Saw” model of tissue fitness: High Mesenchymal Fitness → Suppresses Epithelial Expansion → Maintains Alveolar Homeostasis. Conversely, Low Mesenchymal Fitness→ Releases Epithelial Inhibition → Triggers Aberrant Epithelial Hyper-expansion [4] (Figure 3). This model has profound implications for understanding fibrosis, suggesting that epithelial hyperactivity (bronchiolization) can be a secondary consequence of mesenchymal frailty.

## 4. The Functional Paradox of Cytoplasmic YAP

Traditionally, phosphorylated YAP sequestered in the cytoplasm has been viewed as biologically inert, a waiting room for degradation or nuclear entry. However, emerging evidence challenges this view, attributing active signaling functions to the cytoplasmic pool. This concept is essential for understanding the phenotypic differences between Yap deletion (loss of protein) and Hippo pathway inactivation (increase in both nuclear and cytoplasmic protein).

### 4.1. Cytoplasmic YAP as a Wnt/FGF Inhibitor

Recent studies have elucidated a complex, two-pronged mechanism by which YAP regulates lung epithelial lineage commitment, acting as both a nuclear Hippo effector and a cytoplasmic inhibitor of the Wnt/Fgf10 axis [19].

**The Nuclear Driver (Hippo Inactive):** Integrin-linked kinase (Ilk) normally maintains airway epithelial quiescence by stabilizing Merlin and activating the Hippo pathway, thereby sequestering YAP in the cytoplasm. When Ilk is lost (or during injury), Hippo signaling is inactivated, leading to the nuclear accumulation of YAP. This nuclear YAP directly drives the expression of *Wnt7b* in the epithelium. Secreted Wnt7b then acts on the underlying mesenchyme to induce *Fgf10* expression, which feeds back to the epithelium to promote proliferation and maintain a progenitor state. Thus, Hippo inactivation locks the lung in a high-Wnt/high-Fgf10 amplification loop via a nuclear gain-of-function [19].

**The Cytoplasmic Brake (Hippo Active):** Conversely, cytoplasmic YAP plays an active role in dampening Wnt signaling. Cytoplasmic YAP is an integral component of the β-catenin destruction complex, facilitating the recruitment of β-catenin for degradation. This cytoplasmic “brake” ensures that Wnt signaling remains low during differentiation [26].

### 4.2. Convergence of Genetic Models

This dual mechanism resolves the apparent paradox where two opposing genetic manipulations—Yap knockout (loss of protein) and Ilk/Hippo knockout (gain of nuclear protein)—result in a similar phenotype of stalled differentiation and Wnt hyperactivity.

**Yap Knockout:** The complete loss of YAP protein eliminates the cytoplasmic “brake,” causing the β-catenin destruction complex to fail. This results in the stabilization of β-catenin and spurious activation of Wnt signaling, which prevents differentiation [26].

Ilk/Hippo Knockout: The inactivation of Hippo drives YAP into the nucleus. While this reduces the cytoplasmic pool, the dominant effect is the nuclear YAP-driven expression of *Wnt7b*, which actively ligands the pathway and drives Fgf10 production.

Conclusion: Both models converge on hyperactive Wnt/β-catenin and Fgf10 signaling, leading to an expansion of distal progenitors and a block in maturation. Proper differentiation therefore requires a precise “Goldilocks” balance: sufficient Hippo activity to keep YAP cytoplasmic (suppressing Wnt/β-catenin) while preventing the nuclear accumulation that drives Wnt7b/Fgf10 ligand production [19].

## 5. The Molecular Logic of Alveolar Differentiation and Non-Redundancy of YAP and TAZ

The transition from a proliferating progenitor to a terminally differentiated Alveolar Type 1 (AT1) cell is one of the most critical steps in lung biology. AT1 cells cover 95% of the alveolar surface area and are essential for gas exchange. Recent high-resolution studies have mapped the molecular logic of this transition, identifying a specific requirement for TAZ (as opposed to YAP) [5,11,27].

While YAP regulation in the lung is primarily governed by phosphorylation-dependent shuttling and degradation (the “protein stability switch”), recent evidence indicates that TAZ (WWTR1) is regulated through a distinct transcriptional feed-forward mechanism that functions as a “terminal differentiation lock” for AT1 cells.

**The Initiation (Breaking Stem Cell Quiescence):** The transition from an AT2 stem cell to a differentiated AT1 cell begins with the loss of niche signals. In the homeostatic niche, a single fibroblast provides juxtacrine Wnt signals to a neighboring AT2 cell, maintaining its stemness. This loss of Wnt signaling likely triggers the initial transcription of the *Wwtr1* (TAZ) gene [28].

Unlike YAP, which is constitutively expressed but sequestered in progenitors, TAZ is transcriptionally restricted during early development and in airway and AT2 epithelial cells, becoming robustly expressed only during alveolarization and specifically in AT1 cells [27].

**The Lock (The TAZ-NKX2-1 Positive Feedback Loop):** Once TAZ protein is produced, it enters the nucleus and functions not just as an effector, but as a spatial director for the lung lineage factor NKX2-1. NKX2-1 cannot bind to AT1-specific enhancers on its own; it requires TAZ/TEAD complexes to recruit it to these sites [11]. Critically, since *Wwtr1* itself is an AT1-specific gene, its own enhancer likely requires this same TAZ-NKX2-1 complex for sustained expression. This creates a self-reinforcing positive feedback loop where initial TAZ production leads to nuclear TAZ recruiting NKX2-1 to AT1 enhancers, which drives high-level expression of AT1 genes (*Hopx*, *Ager*) and *Wwtr1* itself. Sustained TAZ production “locks” the cell into the squamous AT1 phenotype. This model explains why AT1 differentiation is stable and why ectopic activation of TAZ (via Hippo pathway deletion) is sufficient to force AT2 cells into the AT1 lineage even without niche exit [6].

A recurring theme throughout this analysis is the necessity to distinguish between YAP and TAZ. They are not interchangeable.

**YAP (Yes-associated Protein):** Essential for early branching morphogenesis and airway progenitor expansion; drives mechanical force generation via *Arhgef17* [20]. Promotes airway basal cell fate and “stemness.” High activity drives proliferation and metaplasia (bronchiolization). Essential for “supercompetitor” status in airway progenitors [5]. Upregulated in aberrant epithelium; drives matrix stiffening via LOX [29].

**TAZ (*WWTR1*):** Crucial for late-stage alveologenesis and alveolar maturation; required for AT1 specification via NKX2-1 feed-forward loop [11]. Required for the differentiation of AT2 cells into AT1 cells. Promotes functional alveolar regeneration [6]. Loss impairs the regeneration of AT1 cells, stalling repair. Drives *TFRC* and iron uptake in fibroblasts [30].

## 6. The Vascular Niche and Pulmonary Hypertension

While much of pulmonary Hippo biology focuses on the epithelium, the pathway is equally critical in the pulmonary vasculature. Dysregulation here is a primary driver of Pulmonary Arterial Hypertension (PAH), a disease defined by vascular remodeling and stiffening.

### 6.1. Non-Canonical Signaling in Vascular Smooth Muscle in Pulmonary Artery Smooth Muscle Cells (PASMCs), the Hippo Pathway Operates Through Distinct, Often Non-Canonical Mechanisms to Drive the Hyper-Proliferation Characteristic of PAH

**The MST1/2-CDC20 Axis:** Unlike the canonical kinase cascade, MST1/2 in PASMCs regulates cell cycle progression via **CDC20**, a co-activator of the Anaphase-Promoting Complex (APC/C). MST1/2 leads to CDC20 stabilization and accumulation in PASMCs, driving aberrant vascular proliferation independent of YAP/TAZ transcriptional activity [31].

**ILK-Hippo Crosstalk:** Integrin-linked Kinase (ILK) acts as a critical upstream regulator in the vasculature. In PAH, LATS1 is inactivated in small pulmonary arteries. This leads to the upregulation of YAP, which increases the production and secretion of fibronectin. Fibronectin subsequently upregulates Integrin-linked kinase 1 (ILK1). ILK upregulation inhibits the Hippo pathway maintaining LATS1 inactivation, leading to nuclear YAP/TAZ retention. This ILK-YAP axis promotes a “senescence-escape” phenotype, allowing vascular cells to proliferate despite metabolic stress [32] (note that ILK affects the Hippo pathway in opposite ways in mesenchyme compared to epithelium).

### 6.2. TAZ and Vascular Calcification Vascular Stiffening in PAH Is Often Exacerbated by Calcification

TAZ has been identified as a specific driver of this pathology via the stabilization of RUNX2, the master osteogenic transcription factor. TAZ binds to RUNX2 and prevents its ubiquitin-mediated degradation. This stabilized RUNX2 complex drives the expression of osteogenic genes in smooth muscle cells, effectively converting them into bone-forming cells [33]. This identifies the TAZ-RUNX2 interface as a specific druggable target for preventing vascular calcification, distinct from general YAP/TAZ inhibition.

### 6.3. Pericytes and Morphogenesis

Beyond smooth muscle, pulmonary pericytes utilize Hippo signaling to coordinate angiogenesis with alveologenesis. Pericyte-specific deletion of YAP/TAZ disrupts the endothelial–epithelial coupling required for septum formation, resulting in simplified alveoli [34]. This underscores that the “vascular niche” is an active structural partner in lung development, not merely a passive supply of oxygen.

## 7. The Alveolar Niche: Regeneration via Cell Competition

The adult lung is quiescent but possesses a robust regenerative capacity. Recent studies have fundamentally reframed regeneration not as a simple differentiation process, but as a competitive fitness landscape governed by the “Myc-Hippo” axis [5].

### 7.1. The “Winner” and “Loser” Paradigm

Warren et al. (2024) [5] demonstrated that cellular fitness in the lung is quantified by intracellular Myc levels, which are tuned by Hippo signaling.

**The “Loser” (Regenerative Fate):** In healthy regeneration, AT2 cells engaging the “loser” program (low Myc/YAP, high TAZ) exit the cell cycle to differentiate into functional AT1 cells. Paradoxically, this is the *constructive* fate. Low Myc levels, enforced by TAZ-mediated inhibition of β-catenin signaling [35] predispose AT2 stem cells to exit the cell cycle and differentiate into functional AT1 cells. This “altruistic” differentiation is required to restore the gas-exchange barrier.

**The “Supercompetitor” (Remodeling Fate):** Conversely, cells expressing high Myc (roughly 2-fold higher or more than neighbors) become “supercompetitors.” Driven by YAP and Myc collaboration, these cells evade terminal differentiation and acquire an aggressive, migratory phenotype. In the context of injury, they do not regenerate alveoli but instead drive *bronchiolization*, the invasion of the alveolar space by bronchial epithelial cells. This establishes a hierarchy where YAP drives the expansion of high-fitness progenitors that can overtake the tissue if not checked by the differentiating influence of TAZ.

### 7.2. The Competitive Void

**Figure 4:**
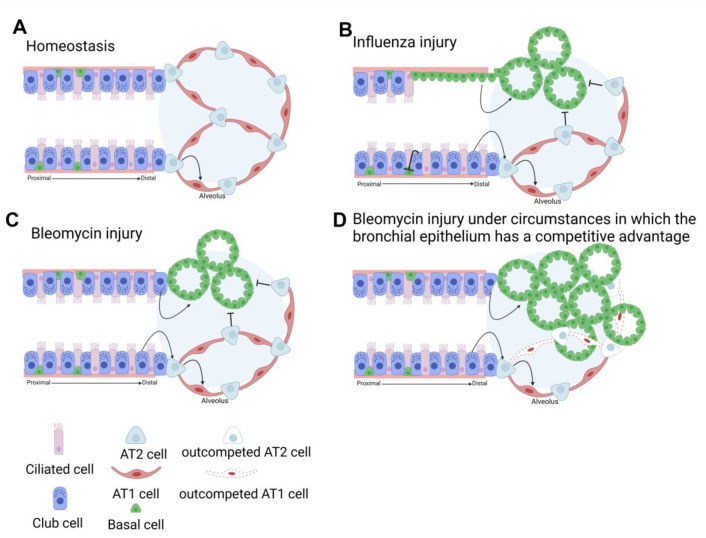
**Bronchial epithelial stem cell response to bleomycin or influenza injury.** (**A**) During homeostasis the alveolar epithelium is maintained by AT2 cells that self-renew and give rise to AT1 cells. (**B**) Upon influenza injury Club cells can regenerate alveolar epithelium if they survive the injury and block basal cell from invading the lung parenchyma. However, if Club cells near the bronchio-alveolar duct junction do not survive the injury, basal like cells derived from rare Trp63^+^ progenitors or serous cells in the airway invade the lung parenchyma to generate basal cell pods which will fill the injured area but do not replace non injured lung tissue. (**C**) Upon bleomycin injury, Club cells can regenerate the alveolar epithelium (AT2/AT1) or drive bronchiolization and give rise to neo-basal cells, which will fill the injured area but do not replace non injured lung tissue. (**D**) However, if bronchial epithelial stem cells acquire a competitive advantage either intrinsic or because AT2 cells have been compromised they will give rise to basal cell that then outcompete these loser AT2 and AT1 cells. Created in BioRender. De langhe, S. https://BioRender.com/8eeovm0 (accessed on 6 January 2026).

Crucially, Warren et al. (2023) [27] demonstrated that loss of fitness in the alveolar compartment can also trigger bronchiolization. When YAP and TAZ are deleted in AT2 cells, these cells fail to differentiate into AT1s (lacking the TAZ-NKX2-1 “lock”). This failure to repair the barrier creates a functional “void” or competitive vacuum [27]. Consequently, airway stem cells (club/basal cells)—even those with baseline fitness—expand into the alveolar space to cover the wound, resulting in bronchiolization. Thus, “honeycombing” is the universal result of competitive imbalance, arising either from the aggression of the airway (high YAP) or the failure of the alveolus (loss of YAP/TAZ) (Figure 4).

### 7.3. Viral-Induced Dysplasia and the IFN\Gamma-YAP Axis

Severe respiratory viral infections (e.g., H1N1, COVID-19) can trigger a maladaptive repair response characterized by the rapid expansion of KRT5+ basal-like cells in the distal alveoli, forming “pods” or scars. Recent studies have elucidated the inflammatory mechanism driving this pathology [36]. Viral infection induces a robust Interferon-γ (IFNγ) response. IFNγ signaling activates the Focal Adhesion Kinase (FAK) and Src kinase axis in p63+ intrapulmonary progenitor cells. This kinase activation induces the nuclear translocation of YAP. The nuclear YAP then drives the proliferation and migration of these dysplastic KRT5+ cells. Crucially, inhibiting YAP during the acute phase prevents pod formation, and inhibiting it in established pods promotes their conversion into beneficial club-like cells, redirecting repair toward functional regeneration [5,36].

### 7.4. Aberrant Epithelial YAP and Matrix Stiffening

In the fibrotic epithelium (the “winner” or “reprogramming” compartment), cells exhibit aberrant, high levels of nuclear YAP activity. These cells, often termed “aberrant basaloid cells” or “intermediate cells,” express markers such as CTGF, AXL, and AJUBA. Critically, YAP in these fibrotic AT2 cells directly regulates the expression of Lysyl Oxidase (LOX), an enzyme responsible for collagen crosslinking [29]. This establishes a vicious cycle: Niche collapse or injury triggers epithelial YAP upregulation (a maladaptive fitness response) → High Epithelial YAP induces LOX secretion → LOX crosslinks collagen, stiffening the matrix → Stiff matrix feeds back to further activate YAP via mechanotransduction (durotaxis pathways) in both fibroblasts and epithelium, perpetuating the cycle.

### 7.5. Age-Dependent Plasticity and Homeostasis

Active Hippo signaling (keeping YAP/TAZ low/cytoplasmic) is required to maintain the AT2 fate in adulthood as inactivation of the Hippo kinases Mst1/2 or Merlin in adult AT2 cells leads to their spontaneous differentiation into AT1 cells [2,5,27]. Furthermore, recent evidence suggests that YAP/TAZ play a role in maintaining epithelial homeostasis by inhibiting goblet cell fate. Deletion of YAP/TAZ leads to airway goblet metaplasia and drives distinct pulmonary inflammatory responses, mediated in part by factors produced by the goblet cells that activate alveolar macrophages [37]. This indicates that basal or cytoplasmic YAP/TAZ activity is required to suppress aberrant differentiation programs even in the absence of overt injury.

## 8. Mechanotransduction: Homeostasis, Controversies, and Technical Limitation

The adult lung is a mechanically dynamic environment, subjected to cyclical strain with every breath. While it is undisputed that mechanical forces influence cell fate, the specific mechanisms and the identity of the mechanosensors in the alveolar compartment have become a subject of intense debate.

### 8.1. The Controversy of Alveolar Epithelial Mechanotransduction

A prominent study by Shiraishi et al. (2023) [38] proposed a model wherein biophysical forces mediated by respiration are the primary sustainers of AT1 cell fate. The authors argued that loss of mechanical strain (via bronchial ligation) or deletion of the mechanosensors Cdc42 and Ptk2 leads to the “reprogramming” of AT1 cells into AT2 cells. They further asserted that nuclear YAP is a specific feature of AT1 cells and is required for their maintenance [38]. However, while *Hopx* is a canonical AT1 marker, the *Hopx-CreERT2* driver line has been shown in multiple independent validations—specifically by Liu et al. (2024) [39]—to be promiscuous or leaky. It labels a substantial fraction of AT2 cells, during homeostasis. Consequently, the “reprogrammed” AT2 cells observed by Shiraishi et al. following loss of tension could represent the proliferation and expansion of *pre-labeled AT2 cells* (which naturally express *Hopx* at low levels or activate the driver transiently) rather than a bona fide conversion of AT1 cells. If the starting population contained labeled AT2s, their expansion in a “relaxed” lung would physiologically mimic reprogramming. Furthermore, the assertion that YAP is the critical maintenance factor for AT1 cells contradicts robust genetic data from other groups. These studies demonstrate that TAZ, rather than YAP, is the essential factor for AT1 differentiation and maintenance [5,6]. High levels of nuclear YAP are characteristically associated with undifferentiated progenitors, aberrant basaloid cells in fibrosis, or hyperplastic AT2 cells—not the quiescent, terminally differentiated AT1 lineage. Genetic deletion of Yap alone does not result in the loss of AT1 cells, whereas deletion of Wwtr1 (TAZ) significantly impairs AT1 generation [5,27]. While Shiraishi et al. provide compelling evidence for mechanical regulation, the use of *Hopx-CreERT2* necessitates caution due to potential lineage labeling issues. Further studies utilizing a more specific *CreERT2* driver or dual-recombinase systems may be required to definitively reconcile the specific roles of YAP versus TAZ in AT1 maintenance or whether YAP/TAZ signaling is even required for AT1 maintenance.

### 8.2. Fibroblast Mechanosensing and Durotaxis

In the mesenchymal compartment, the mechanotransduction roles of YAP and TAZ are less controversial and critically important for fibrosis. A critical component of fibrotic progression is durotaxis—the migration of cells along gradients of extracellular matrix stiffness. Recent work has elucidated that this is mediated by the interaction between Focal Adhesion Kinase (FAK) and paxillin [40]. This mechanosensory module links stiffness cues to transcriptional programs via YAP signaling. Disrupting the FAK–paxillin interaction blocks durotaxis and attenuates fibrosis, establishing mechanical YAP activation as a pathogenic driver [40]. In the mesenchymal compartment, YAP and TAZ function as critical regulators of contractility and matrix stiffness sensing. While they are often redundant, their combined activity is essential. Single knockdown of YAP or TAZ in lung fibroblasts has limited effects on contractile gene expression. However, combined knockdown significantly reduces the expression of ACTA2 (α-SMA), CNN1, and TAGLN, compromising the cell’s ability to generate traction forces [41]. The mechanotransduction signaling axis in fibroblasts involves G-protein coupled receptors (GPCRs). Activation of Gαs-coupled receptors leads to cAMP generation, which acts through EPAC1/2 and RAP2C to activate MAP4K7 [42]. This kinase cascade ultimately phosphorylates LATS1/2, leading to YAP/TAZ inactivation. This pathway identifies the cAMP-EPAC axis as a potent therapeutic target: elevating cAMP or activating specific dopamine receptors can suppress the aberrant mechanosignaling and YAP nuclear localization that drives fibroblast activation in fibrosis.

## 9. The Mechanobiology of Fibrosis: Niche Collapse and Bronchiolization

Idiopathic Pulmonary Fibrosis (IPF) is characterized by the replacement of alveoli with scar tissue and “honeycomb cysts” (aberrant airways). The integration of recent findings suggests this pathology can also be driven by a failure of inter-tissue cell competition.

### 9.1. The “Niche Collapse” Model

Klinkhammer et al. (2025) propose that bronchiolization may not begin in the epithelium, but with a loss of fitness in the mesenchyme [4].

**Alveolar Fibroblast (AF1) Fitness:** The maintenance of the alveolar niche (AF1 cells) is an active process dependent on Yap/Taz and Snail1/2.

**The Collapse:** If AF1s lose fitness (e.g., due to aging or genetic mutation of Yap/Taz, Snai1/2 or Myc), they undergo apoptosis. This “Niche Collapse” removes the essential support for AT2 cells, leading to secondary epithelial death. Fgf10 is a major niche factor essential for AT2 maintenance [43]. While transient reduction in YAP/TAZ normally induces fibroblasts to secrete Wnt4 to drive repair, the apoptotic elimination of these cells results in the total loss of this critical signal. The absence of mesenchymal Wnt4 arrests AT2 proliferation and impairs alveolar repair, confirming it as a critical molecular currency of the niche [44].

**The Reaction:** The remaining airway stem cells sense this “competitive vacuum.” Released from the suppressive signals of the healthy niche, they initiate a pathological expansion [4,5].

### 9.2. Bronchiolization as De Novo Submucosal Gland Development

**Figure 5:**
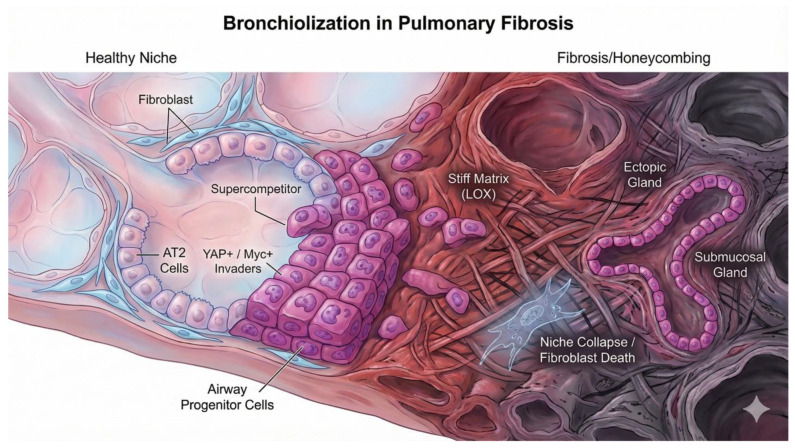
**Niche collapse and the mechanics of bronchiolization in pulmonary fibrosis.** A landscape cross-section comparing a healthy alveolar niche (**Left**) with a fibrotic lesion (**Right**). In the healthy lung, alveolar fibroblasts maintain epithelial quiescence. In Idiopathic Pulmonary Fibrosis (IPF), the apoptosis of these fibroblasts (“Niche Collapse”) creates a competitive void. High-fitness airway progenitors (magenta), functioning as YAP+/Myc+ “supercompetitors,” migrate into this void. These invaders differentiate into ectopic, gland-like structures (“Honeycombing”) that morphologically resemble bronchial submucosal glands, a process perpetuated by stiffness-dependent YAP activation via the LOX-crosslinked matrix. Created with Nano Banana Pro.

The resulting epithelial invasion—bronchiolization—is not random metaplasia. Warren et al. (2024) [5] identified that the invasive cells at the leading edge of these lesions express a unique signature (Krt5+/Sox9+/Acta2+/Myc+). This phenotype is identical to the myoepithelial cells (MECs) of submucosal glands (SMGs). Thus, the “honeycomb cysts” of IPF represent a desperate, high-fitness attempt by the lung to “seal” the injury by initiating a program of de novo submucosal gland development in the distal lung, driven by YAP/Myc “supercompetitors” filling the void left by the collapsed niche [5] (Figure 5).

### 9.3. The Snail-YAP Rheostat and Ferroptosis

The fate of the mesenchymal niche is regulated by a molecular switch involving Snail1/2 (Snail/Slug) and Hippo signaling [41].

**Homeostasis:** Snail1/2 sequesters YAP/TAZ, preventing them from binding TEAD/Myc. This promotes an adipogenic, niche-supportive AF1 phenotype.

**Fibrosis:** When this sequestration fails, YAP/TAZ bind TEAD/Myc, instructing a myogenic, pro-fibrotic myofibroblast fate.

**Ferroptosis:** Recent studies have added a metabolic dimension to this switch. The TAZ-TEAD program in fibroblasts elevates the expression of the *Transferrin Receptor* (*TFRC*), increasing iron uptake. This iron accumulation promotes myofibroblast conversion but also renders the cells susceptible to *ferroptosis*. However, simultaneous metabolic reprogramming often protects these activated fibroblasts from ferroptotic death, allowing them to persist. This TAZ-iron-ferroptosis axis represents a novel vulnerability in the fibrotic mesenchyme [30].

## 10. Transitional States and Malignancy: The Cancer Connection

The relevance of Hippo signaling extends to lung cancer, specifically linking the “supercompetitor” state to malignancy.

### 10.1. SCLC: MYC Paralogs Dictate Evolutionary Trajectories and “Supercompetitor” Status

**Figure 6:**
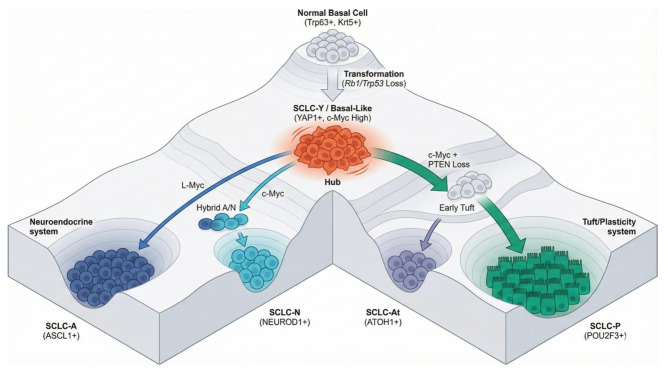
**Waddington epigenetic landscape model of SCLC lineage plasticity from a basal cell of origin.** The landscape illustrates the hierarchical differentiation of Small Cell Lung Cancer (SCLC) subtypes from a **Normal Basal Cell** ancestor (*Trp63*+, *Krt5*+). Transformation via the obligate loss of *Rb1* and *Trp53* generates a highly plastic, basal-like progenitor state (**SCLC-Y**), characterized by YAP1 expression and high c-Myc activity. From this central “Hub,” lineage trajectories bifurcate based on specific genetic drivers: **Neuroendocrine System** (**Left**): Genetic pressure from *L-Myc* (*MYCL*) restricts cells to the classic **SCLC-A** (*ASCL1*+) fate. Alternatively, *c-Myc*-driven plasticity facilitates a transition through a **Hybrid A/N** state to the **SCLC-N** (*NEUROD1*+) fate. **Tuft/Plasticity System** (**Right**): The cooperation of *c-Myc* overexpression and **PTEN loss** drives cells away from neuroendocrine fates toward an **Early Tuft** intermediate. This lineage culminates in the **SCLC-P** (Tuft, *POU2F3*+) fate or branches into the **SCLC-At** (*ATOH1*+) fate. The SCLC-Y state functionally mirrors the **“supercompetitor” aberrant basaloid cell** observed in pulmonary fibrosis. Both states are driven by high **YAP/c-Myc** activity. Created with Nano Banana Pro. **The L-MYC vs. c-MYC Dichotomy:** The classic neuroendocrine subtype (SCLC-A) is driven by *L-MYC* (*MYCL*) amplification. These tumors retain a high neuroendocrine identity (ASCL1+) and lower metastatic potential compared to variants. In stark contrast, the variant, non-neuroendocrine subtypes (SCLC-N, SCLC-P, and SCLC-Y) are typically driven by *c-MYC* (*MYC*) amplification or overexpression.

Small Cell Lung Cancer (SCLC) has historically been viewed as a homogeneous neuroendocrine disease. However, recent genetic modeling has revealed that distinct subtypes are driven by mutually exclusive MYC family paralogs, which dictate the tumor’s evolutionary trajectory and its resemblance to regenerative states [45] (Figure 6).

**The c-MYC Evolutionary Trajectory:** High levels of c-MYC do not merely drive proliferation; they force a temporal dedifferentiation. Research by Ireland et al. (2025) [45] demonstrated that c-MYC drives cells along a specific plasticity trajectory: from an YAP1+ (SCLC-Y) state → NEUROD1+ (SCLC-N) → ASCL1+ (SCLC-A) state. Thus, SCLC-Y represents an early-stage, c-MYC-driven, non-neuroendocrine phenotype that has shed its lineage identity.

**SCLC-Y as the “Supercompetitor” (Mesenchymal/SCMC):** The SCLC-Y subtype is defined by the co-expression of *high YAP and high c-MYC* [45]. This profile mirrors the “Supercompetitor Myoepithelial Cell” (SCMC) described in pulmonary fibrosis [5]. Just as SCMCs (Krt5+/Sox9+/Myc+/YAP+) outcompete alveolar cells to drive bronchiolization in fibrosis, SCLC-Y cells utilize this high-fitness, high-metabolism state to remodel the tumor microenvironment, resist chemotherapy, and drive metastasis.

**SCLC-P (Tuft Branch):** While also driven by *High c-MYC*, the SCLC-P (POU2F3+) subtype represents a distinct branch off this trajectory, specifically catalyzed by *PTEN loss* (PI3K/AKT activation). Recent research utilizing genetically engineered mouse models has identified the *basal cell* as the likely origin for the POU2F3+ tuft-like SCLC subtype [45]. While originating from YAP+ basal cells, SCLC-P tumors typically downregulate YAP1 as they differentiate into the tuft lineage, distinguishing them from the YAP-retaining SCLC-Y supercompetitors. Interestingly basal cell pods that appear after H1N1 injury also feature Tuft cells [46,47,48].

### 10.2. NSCLC: A Division of Labor

In Non-Small Cell Lung Cancer (NSCLC), YAP and TAZ exhibit a distinct “division of labor.” While often co-expressed, they regulate non-identical transcriptional programs. YAP preferentially regulates genes associated with cell cycle progression and division, acting as the primary driver of tumor growth. In contrast, TAZ preferentially regulates genes associated with extracellular matrix interaction, cell adhesion, and epithelial–mesenchymal transition (EMT), thereby driving migration and metastasis [49]. This underscores the necessity of distinguishing between the two paralogs when designing therapeutic strategies, as inhibiting one may not fully abrogate the oncogenic potential of the other.

### 10.3. AP-1 Transcription Factors in Transitional States

Recent multi-omic profiling has identified Activator Protein-1 (AP-1) transcription factors (FOS, FOSB, and JUNB) as central regulators of the injury-induced transitional AT2 cell state (often termed DATP or PATS). Upon viral injury, JUNB and FOSB accumulate in these transitional cells and, along with constitutive FOS, drive the induction of CLDN4, promote AT2 cell dispersion, and initiate senescence signaling toward fibroblasts. This AP-1 activation is not merely a repair response but shares a gene regulatory logic with tumorigenesis. AP-1 activation is also observed in mouse AT2 cells expressing oncogenic Kras and in transitional cells within human lung adenocarcinoma lesions. This suggests that the regenerative plasticity of the lung, if unchecked or dysregulated (e.g., by sustained YAP activity, which often cooperates with AP-1), can predispose the tissue to neoplastic transformation [14].

## 11. Therapeutic Frontiers: Modulating Fitness and Mechanics

Understanding fibrosis as a disease of dysregulated competition and niche collapse opens new therapeutic avenues (Table 1).

### 11.1. Restoring Niche Fitness

Instead of just killing fibroblasts, we may need to “rescue” the fitness of the alveolar niche to prevent epithelial bronchiolization.

**Snail Stabilizers:** Since Snail1/2 are protective in fibroblasts (sequestering YAP), small molecules that stabilize Snail proteins could maintain the protective AF1 state [41].

**Omentin-1:** The adipokine Omentin-1 has been shown to induce lipogenic differentiation in mechanically activated fibroblasts via the *PKM2/YAP/PPARγ* pathway. Omentin-1 increases levels of Fructose-1,6-bisphosphate (FBP), which binds to Pyruvate Kinase M2 (PKM2), preventing it from interacting with YAP. This decoupling promotes the reversion of myofibroblasts to a lipid-storing phenotype, promoting fibrosis resolution [50].

### 11.2. Approved Drugs as Hippo Modulators

Perhaps the most significant finding for current patients is that Nintedanib, one of the two approved antifibrotics, functions partly as a Hippo pathway inhibitor. By inhibiting TBK1 (TANK-binding kinase 1), an upstream regulator that stabilizes YAP in response to stress, Nintedanib dampens the mechanotransductive response [51]. This suggests that combining Nintedanib with agents that target the pathway at a different node (e.g., Statins targeting the Mevalonate-Rho axis to block YAP nuclear entry) could produce synergistic effects.

## 12. Conclusions

The integration of classical developmental biology with the new paradigms of cell competition and niche collapse provides an integrative framework of lung fibrosis. The disease is not merely a scarring reaction, but a shift in the competitive equilibrium of the tissue. Health is maintained by a “loser” state in the epithelium (TAZ-driven differentiation) and a “high fitness” state in the mesenchyme (Snail-protected AF1s) [4,5,6,27]. Disease is triggered by mesenchymal niche collapse or epithelial injury, leading to the emergence of “supercompetitor” airway cells (YAP/Myc-driven) that remodel the lung in a misguided attempt to seal the wound. Future therapies must move beyond broad suppression of inflammation and aim to restore this competitive balance—boosting the fitness of the alveolar niche while curbing the aggression of the airway invaders. The identification of novel axes such as the TFRC-ferroptosis pathway in fibroblasts and the AP-1 network in epithelial transition provides tangible targets for the next generation of antifibrotics.

## Figures and Tables

**Table 1:** Therapeutic avenues to target dysregulated competition and niche collapse.

Target/Node	Mechanism of Action	Potential Clinical Application
Nintedanib	Inhibits TBK1, preventing YAP stabilization	Existing antifibrotic; reduces mechanotransduction
Snail Stabilizers	Stabilizes Snail1/2 in fibroblasts; sequesters YAP	Preventing “Niche Collapse” in early fibrosis
Omentin-1	Decouples PKM2/YAP via metabolic modulation	Reverting myofibroblasts to lipogenic phenotype
Statins	Inhibits HMG-CoA reductase (Mevalonate pathway)	Blocking RhoA-mediated nuclear entry of YAP

## Data Availability

No new data were created or analyzed in this study.
